# PD154 Horizon Scanning Report Identifying Technologies For Pediatric Neurological Trauma

**DOI:** 10.1017/S0266462324003866

**Published:** 2025-01-07

**Authors:** Chizoba Oparah, Claire Eastaugh, Kate Lanyi, Amy Hussain, James Woltmann, Fiona Pearson, Madeleine Still, Emily Brush, Niina Kolehmainen

## Abstract

**Introduction:**

Children and young people comprise one quarter of the UK’s population. Neurological trauma (e.g., traumatic brain injury and spinal cord injury) is one of the most common causes of death and disability in this population group, yet health technology innovations for these patients lag behind those for adults.

**Methods:**

Using the horizon scanning methodologies developed by the Innovation Observatory, systematic searches were performed to identify registered clinical trials, published funding awards, and news articles that focused on innovative devices and digital and diagnostic health technologies developed for use in children and young people (from 28 days after birth up to and including 18 years of age) with actual or suspected neurological trauma. The search results were screened for relevance, and key information on the included technologies was extracted and summarized.

**Results:**

Twenty-nine technologies were identified, of which 10 were commercially available. The majority were developed in the UK or the USA. Overall, the development pipeline was evenly split amongst technologies considered to be a device (37%), digital (34%), or diagnostic (29%). Most technologies were intended for use across settings by healthcare professionals, either for initial onsite assessment, for in-hospital management, or for rehabilitation in hospital or in the community.

**Conclusions:**

Results from this horizon scan show that development of technologies for pediatric neurological trauma is currently limited, with only a small number of the technologies being developed covering an area of unmet need. To complement the horizon scan, we also sought stakeholder insights on medical technologies for this population group. The combined results and final conclusions will be shared in a future publication.

